# Gastrodin Ameliorates Cognitive Dysfunction in Diabetes Rat Model via the Suppression of Endoplasmic Reticulum Stress and NLRP3 Inflammasome Activation

**DOI:** 10.3389/fphar.2018.01346

**Published:** 2018-11-22

**Authors:** Tianyuan Ye, Xiangbao Meng, Yadong Zhai, Weijie Xie, Ruiying Wang, Guibo Sun, Xiaobo Sun

**Affiliations:** ^1^Beijing Key Laboratory of Innovative Drug Discovery of Traditional Chinese Medicine (Natural Medicine) and Translational Medicine, Institute of Medicinal Plant Development, Chinese Academy of Medical Sciences, Peking Union Medical College, Beijing, China; ^2^Key Laboratory of Bioactive Substances and Resource Utilization of Chinese Herbal Medicine, Ministry of Education, Beijing, China; ^3^Key Laboratory of Efficacy Evaluation of Chinese Medicine Against Glycolipid Metabolic Disorders, State Administration of Traditional Chinese Medicine, Beijing, China

**Keywords:** cognitive dysfunction, diabetes mellitus, NLRP3 inflammasome, endoplasmic reticulum stress, apoptosis

## Abstract

Patients with diabetes mellitus (DM) are at high risk for cognitive dysfunction. Endoplasmic reticulum stress (ERS) and inflammation play crucial roles in DM. Gastrodin (Gas), the main component of *Gastrodia elata*, possesses anti-oxidative stress, anti-inflammatory, and neuroprotective effects. This present study aims to investigate whether Gas could ameliorate cognitive dysfunction in DM and to explore its underlying mechanisms. Rats with streptozotocin-induced type 2 DM were used in this study. After administration of Gas for 5 weeks, the levels of total cholesterol (TC), triglyceride (TG), low density lipoprotein cholesterol (LDL-C) and high density lipoprotein cholesterol (HDL-C) in serum, TNF-α, IL-1β, MDA and SOD in the hippocampus were measured. Morris water maze, hematoxylin and eosin (HE) and Nissl staining were performed to assess the effects of Gas on cognitive function and hippocampal neuronal apoptosis. Protein levels of GLUT3, brain derived neurotrophic factor (BDNF), GRP78, PERK, P-PERK, TXNIP, ASC, NLRP3, CHOP, Bcl-2 and Bax were measured by using Western blot. The results showed that Gas could improve hyperglycemia and dyslipidemia in DM rats, as the levels of TC, TG LDL-C in serum were decreased. TNF-α, IL-1β, MDA contents in the hippocampus were decreased, and SOD contents was increased in the hippocampus of DM rats. Inflammation, oxidative stress, ERS, and apoptosis were observed in the hippocampus of DM rats, accompanied with decreased expression of BDNF and GLUT3. Gas improved the cognitive deficits caused by diabetes and inhibited inflammation, oxidative stress, ERS, and apoptosis in the hippocampus. Furthermore, Gas substantially increased the expression of GLUT3, and inhibited hippocampal ERS and ERS-mediated apoptosis. Additionally, Gas increased the expression of BDNF and decreased the activation of NLRP3 inflammasome. These results suggested that by inhibiting ERS and NLRP3 inflammasome activation and increasing the expression of BDNF and GLUT3, Gas exhibits neuroprotective effects against cognitive dysfunction in DM.

## Introduction

Type 2 diabetes (T2D), a kind of chronic metabolic disease, has now emerged as a one of the most serious health problems across the world, especially in developing countries. Central effects of T2D include brain insulin resistance, glucose dysmetabolism, changes in autophagic pathway, neuronal death, and cognitive dysfunction, ultimately increase the risk of neurodegenerative diseases (Candeias et al., 2017). Epidemiological investigations suggest that diabetes mellitus (DM) patients have more risks suffering from cognitive dysfunction compared with healthy subjects (Degen et al., 2016). However, the mechanisms underlying cognitive dysfunction in T2D remain poorly understood.

The precise link between T2D and neurodegeneration remains unclear; however, inflammatory pathway may play crucial role in the development of this kind of disease (Ribe and Lovestone, 2016). As a pro-inflammatory cytokine, the level of IL-1β is remarkably increased in the hippocampus of T2D rats in a previous study (Sun et al., 2017). The maturation of IL-1β is mediated by NLRP3 inflammasome, this kind of inflammasome contains a recognition receptor (NLRP3), an apoptosis-associated speck-like protein containing a card (ASC), and an effector molecule (caspase-1) (Qi et al., 2018). NLRP3 inflammasome is activated in the neurons of hippocampus in db/db mice according to a previous study (Zhai et al., 2018). Recent study showed that NLRP3 inflammasome can be activated by high fat diet-induced hippocampal endoplasmic reticulum stress (ERS), subsequently causing the secretion of IL-1β (Cai et al., 2016). Endoplasmic reticulum (ER) is an intracellular organelle known as a factory where membrane and secretory proteins are assembled (Wang et al., 2016). In mammalian cells, the central proteins involved in initiating ERS are inositol-requiring enzyme 1α (IRE-1α), activating transcription factor 6 (ATF6), and double-stranded RNA dependent protein kinase-like ER kinase (PERK) (Zhang and Kaufman, 2008). GRP78 primarily regulates the initiation of the UPR by its direct interactions with the signal transducing sensor (Sprenkle et al., 2017). The ERS has recently gained considerable attention, and ERS-initiated apoptosis and inflammation have been implicated in cognitive dysfunction secondary to T2D (Wang et al., 2016). UPR signaling mediates cell from pro-survival to pro-apoptosis through the transcriptional induction of C/EBP homologous protein (CHOP), and the increase of Bax/Bcl-2-dependent pathways (Kim et al., 2008). However, the mechanisms underlying ERS on NLRP3 inflammasome in the hippocampus of DM rats remains poorly understood. Besides the ERS and NLRP3 signaling mechanisms, the ER can also regulate the maturation of brain-derived neurotrophic factor (BDNF), which is very crucial in synaptic plasticity in the hippocampus. However, how excessive ERS causes the disruption in the process of BDNF maturation is still uncertain.

**FIGURE 1:**
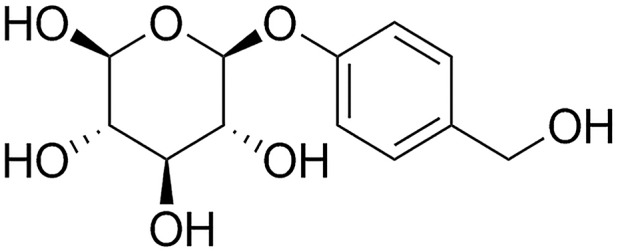
Chemical structure of Gas; molecular weight is 286.3; molecular formula is C_13_H_18_O_7_.

Gastrodin (Gas, the molecular structure is shown in Figure 1) is one of the main components in the rhizome of *Gastrodia elata*. The effects of Gas on central nervous system include modulating neurotransmitters, suppressing microglial activation, anti-oxidative stress, anti-inflammation, and up-regulating neurotrophins (Liu et al., 2018). In an AD mouse model, Gas can decrease β-secretase level by suppressing the protein kinase/eukaryotic initiation factor-2α (PKR/eIF2 α) pathway (Zhang et al., 2016). Moreover, Gas can increase the contents of HO-1, GSH, SOD, and ERK1/2 phosphorylation, Nrf2 nuclear translocation can also be increased in the striatum, showing that by upregulating ERK1/2-Nrf2 pathway, Gas can protect the midbrain of MPTP intoxicated mice (Wang et al., 2014). These above researches suggests the multiple potential effects of Gas on neurodegenerative diseases (Sun et al., 2012). However, whether Gas can ameliorate cognitive dysfunction in diabetes mellitus remains unclear.

There are no investigations concerning about whether Gas can ameliorate cognitive deficits in diabetic rats. In our study, we measured fasting blood glucose (FBG) and body weight weekly and examined behavioral changes and hippocampal inflammatory cytokines of DM rats. To investigate the neuroprotective effects of Gas and explore its mechanisms in DM-induced cognitive dysfunction, we measured the expression levels of protein in ERS, inflammatory and apoptotic signaling pathways. Based on the results, Gas inhibited hyperglycemia-induced ERS and NLRP3 inflammasome activation and increased BDNF expression in the hippocampal neurons. Therefore, Gas can be used to prevent and treat cognitive dysfunctions secondary to DM.

## Materials and Methods

### Reagents

Gastrodin (Gas, its molecular weight is 286.28, CAS NO: 62499-27-8, purity>99%) was obtained from Shanghai Winherb Medical Science Company (Shanghai, China). Streptozotocin (STZ) was obtained from Sigma-Aldrich (St. Louis, MO, United States). The enzyme-linked immunosorbent assay (ELISA) kits for detecting the rat IL-1β and TNF-α were obtained from DAKEWEI (Shenzhen, China). The kits for determining MDA and SOD were obtained from Nanjing Jiancheng Institute (Nanjing, China). The kits for detecting triglyceride (TG), total cholesterol (TC), low density lipoprotein cholesterol (LDL-C) and high density lipoprotein cholesterol (HDL-C) were purchased from Biosino Biotechnology & Science Inc. (Beijing, China). Primary antibodies against NLRP3, BDNF, TXNIP, and GLUT3 were from Abcam (Cambridge, MA, United States). Primary antibodies against CHOP, PERK, P-PERK, ASC, Bax, and Bcl-2 were provided by Santa Cruz Biotechnology (Santa Cruz, CA, United States).

### Animals and Treatments

The animals were purchased from Vital River Laboratory Animal Technology Co. Ltd. (Beijing, China). Six-week-old male Sprague-Dawley (SD) rats weighting 200–220 g were maintained at room temperature (25°C) and in a 12-h light and 12-h dark cycle and allowed to have access to water and chow. All laboratory procedures were performed according to the Research Ethics Committee of the Chinese Academy of Medical Sciences and Peking Union Medical College, Beijing, China (SCXK 2014-0001).

The experimental rats were randomly assigned to two groups: control group with standard diet (*n* = 10) and DM group (*n* = 55). DM group rats were given a high-fat and high-sucrose diet consists of 24% protein, 41% carbohydrate and 24% fat. Rats were fed for 4 weeks. Once insulin resistance was developed, DM was induced by intraperitoneal injection of 35 mg/kg STZ. Rats with FBG levels higher than 11.1 mM after 7 days were considered as DM. After the induction of DM for 4 weeks, the DM rats were administered with Gas (50 or 100 mg/kg) once a day for 5 weeks.

**FIGURE 2:**
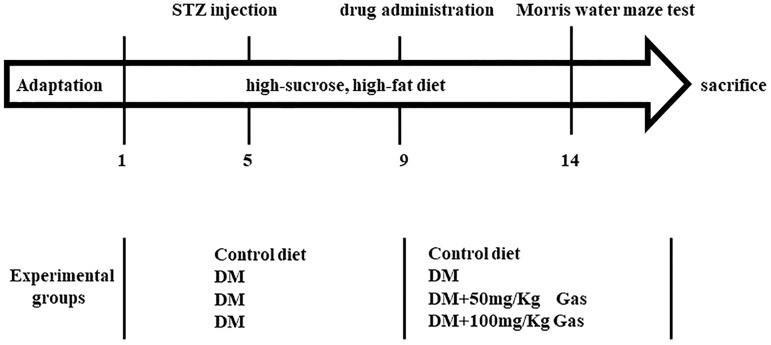
Summary of the experimental design.

Body weights and FBG were monitored once per week. On the fifth week, animals were trained to perform learning and memory tasks in Morris water maze (MWM) for five consecutive days. At the end of the behavioral study, the hippocampus was harvested for subsequent biochemical and morphological studies (Figure 2).

### Oral Glucose Tolerance Test (OGTT) and Insulin Tolerance Test (ITT)

Oral Glucose Tolerance Test was performed according to previous methods with minor modifications. After the rats were fasted for 12 h, OGTT was conducted by intragastric administration of glucose solution (2 g/Kg). Blood glucose levels were detected 0, 30, 60, 90 and 120 min following the glucose load.

After 6 h of fasting, the rats were administered intraperitoneally with 0.75 U/Kg of human insulin (Wanbang, China). Blood glucose levels were detected 0, 30, 60, 90 and 120 min following insulin injection.

### Morris Water Maze (MWM) Test

Spatial learning and memory function of rats were measured by MWM test. The water pool was 120 cm in diameter and 60 cm in height, filled with 20–24°C non-toxic black water (45 cm in depth). The pool was located in a quiet room with visual cues that could be seen by rats for orientation. A black platform 10 cm in diameter and 2 cm below the surface of the water, was placed in the center of one quadrant, and remained in the same quadrant during the entire experiment. A computer with a management system was used to record the performance of rats. The trainings were performed thrice daily for four consecutive days with 1 h interval. In this 4-day training test, rats were softly put into the water at one of the four different starting positions facing the pool wall and then swam freely to find the platform. The spent time of each rat to reach the submerged platform (escape latency) was recorded. After the rat reached the platform, it was allowed to stay on the platform for 15 s. If the rat can’t reach the platform in 90 s, the test was ended and its escape latency was recorded as 90 s, it was then guided to stay on the platform for 30 s. On the fifth day, the platform was removed, the rat was allowed to swim freely for 90 s. The number of rats crossing the platform and escape latency were measured. All the trials were performed between 8:00 a.m. and 5:00 p.m.

### Preparation of Tissue Sample

Overnight fasted rats were deeply anesthetized with 1% sodium pentobarbital (40 mg/Kg). Blood samples were collected and kept at room temperature for 30 min to allow the samples to clot. Afterward, these samples were centrifuged to collect the serum, and then stored at -80°C until analysis.

In each group, a total of three rats were transcardially perfused with 4% paraformaldehyde. Brains were softly removed, and then immerged in the fixative solution for 24 h. They were then used for hematoxylin and eosin (HE) and Nissl staining. The remaining rats were decapitated and the hippocampus were softly isolated from the brain and washed with 0.9% cold saline for three times. The tissues were stored at -80°C until analyzed. The right side hippocampus were used for Western blot.

### Measurement of Serum Lipids

The contents of TC, TG, LDL-C and HDL-C from serum were measured by using Hitachi 7600 Automatic Biochemistry Analyzer (Tokyo, Japan).

### Measurement of IL-1β, TNF-α, SOD and MDA

The concentrations of IL-1β and TNF-α in hippocampal supernatants were separately determined using ELISA kits according to the manufacturer’s instructions. The absorbance was determined by a microplate reader (SpectraFluor, TECAN, Sunrise, Austria) at 450 nm.

The activities of SOD and MDA in hippocampal supernatants were separately measured by kits according to the manufacturer’s instructions.

### Hematoxylin and Eosin (HE) and Nissl Staining

In this experiment, four rats in each group were used to analyze HE and Nissl staining. Brain specimens were immersed in 4% paraformaldehyde for 24 h after being extracted. Afterward, the brains were embedded in paraffin, and tissue sections (4–6 μm) were obtained for HE staining. For Nissl staining, we conducted the experiments according to a previously described method. Light microscopy was used to observe the images (Life Technologies).

### Western Blot

Total protein of hippocampus were extracted by using RIPA buffer, supplemented with protease inhibitors. The tissue were extracted on ice and the supernatant was collected after centrifuged for 15 min at 4°C. Protein concentration was measured by the BCA assay kit (Beyotime Institute of Biotechnology, China). Then, the protein samples were heated at 95°C for10 min. Equal amounts of protein (30 μg) were loaded onto 8–12% SDS polyacrylamide gels and separated via electrophoresis at 80 V for 120 min. The proteins were transferred onto NC membranes and then blocked by 5% non-fat milk at room temperature for 2 h. The membranes were incubated overnight at 4°C overnight with different antibodies including TXNIP (1:1,000), NLRP3 (1:1,000), ASC (1:200), GRP78 (1:200), PERK (1:200), P-PERK (1:200), CHOP (1:200), GLUT3 (1:2,000), BDNF (1:1,000) and β-actin (1:1,000). The next day, membranes were washed in TBST for three times and incubated with the relevant secondary antibodies for 2 h. Subsequently, the NC membranes were washed in TBST for three times and then visualized with enhanced chemiluminescence solution. Images were obtained using Molecular Imager Lab (Bio-Rad, United States).

### Statistical Analysis

Experimental data in this research were expressed as means ± SD. Differences between groups were determined by one-way analysis of variance (ANOVA) using SPSS 17 (SPSS, United States), followed by least significant difference post hoc comparison test. Indexes in MWM tests were analyzed by repeated-measure two-way ANOVA. *P* < 0.05 was regarded as statistically significant.

## Results

### Effects of Gas on Body Weight and FBG Level in DM Rats

**FIGURE 3:**
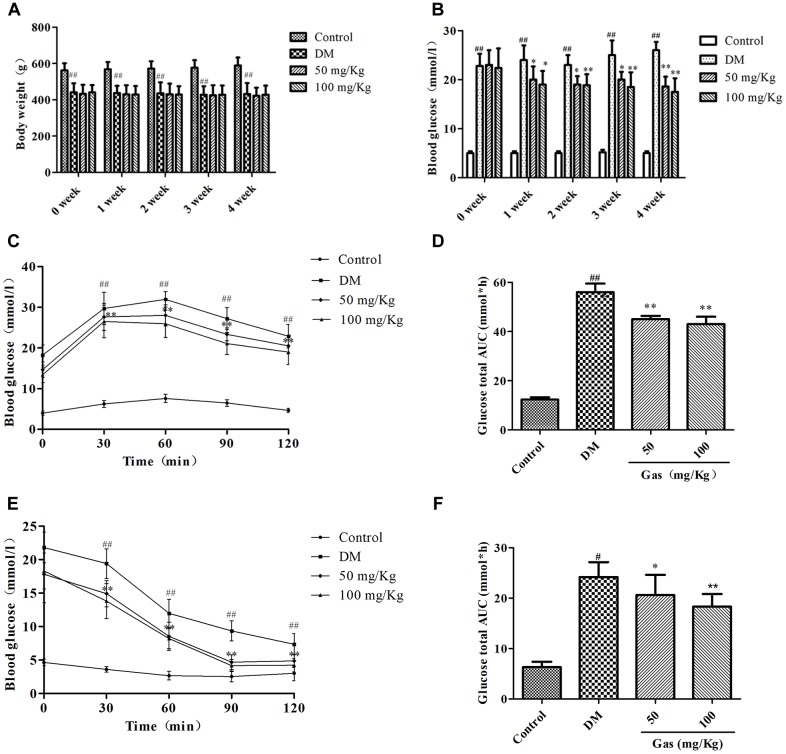
Gas improves insulin resistance in DM rats. **(A)** Body weight of rats in each group during 5 weeks of treatment. **(B)** Fasted blood glucose level of rats in each group during 5 weeks of treatment. **(C)** Curve of blood glucose level in OGTTs. **(D)** Glucose total AUC in OGTTs. **(E)** Curve of blood glucose level in ITTs. **(F)** Glucose total AUC in ITTs. All data are expressed as means ± SD for 10 rats in each group. ^#^*P* < 0.05,^##^*P* < 0.01, compared with the control group. ^∗^*P* < 0.05, ^∗∗^*P* < 0.01, compared with the DM group.

As shown in Figure 3A, body weight of the DM rats were significantly decreased when compared with those in the control group (*P* < 0.01), and no apparent differences were noted in body weight between rats from Gas groups and DM group until the end of the treatment. As shown in Figure 3B, FBG in DM group was higher than the control group (*P* < 0.01), Gas (50, 100 mg/Kg) effectively lowered the FBG levels of DM rats to some extent but did not restore them to normal levels (*P* < 0.05, *P* < 0.01).

### Gas Treatment Improved Glucose Tolerance in DM Rats

In accordance with our previous studies, blood glucose was detected at different time points. As shown in Figure 3C, FBG levels were obviously higher in DM rats compared with those in control group (*P* < 0.01), Gas treatment could decrease their FBG level to some extent. Glucose total area under the curve (AUC) of the DM rats was significantly higher than that in the control group (*P* < 0.01). Moreover, the glucose total AUC of Gas-treated rats was reduced significantly compared with that in DM rats (*P* < 0.05, *P* < 0.01) (Figure 3D). Furthermore, rapid removal of blood was clearly different in Gas-treated rats compared with DM rats during ITT (*P* < 0.05, *P* < 0.01) (Figure 3E). Additionally, as shown in Figure 3F, AUC of the Gas groups in ITT was remarkably lower than the DM group (*P* < 0.05, *P* < 0.01).

### Gas Treatment Improved Dyslipidemia in DM Rats

**FIGURE 4:**
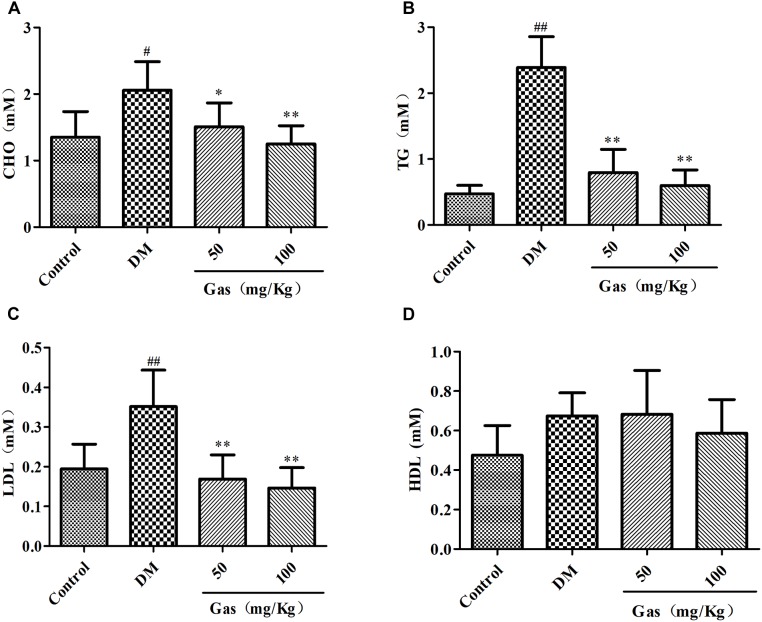
Gas improves dyslipidemia in DM rats. **(A)** Levels of TC in serum samples of rats after 5 weeks of treatment. **(B)** Levels of TG in serum samples of rats after 5 weeks of treatment. **(C)** Levels of LDL-C in serum samples of rats after 5 weeks of treatment. **(D)** Levels of HDL-C in serum samples of rats after 5 weeks of treatment. Values are represented as means ± SD for 10 rats in each group. ^#^*P* < 0.05,^##^*P* < 0.01, compared with the control group. ^∗^*P* < 0.05, ^∗∗^*P* < 0.01, compared with the DM group.

As results shown in Figures 4A–C, large increase of TC (1.35 mM), TG (0.47 mM) and LDL-C (0.19 mM) contents in serum were observed in DM group (respectively, 2.06, 2.39, and 0.35 mM) when compared with the control subjects (*P* < 0.05, *P* < 0.01). However, after Gas treatment for 5 weeks, the expression levels of TC, TG and LDL-C were significantly decreased (*P* < 0.05, *P* < 0.01). However, we didn’t find significant change of HDL-C among these groups. (Figure 4D).

### Gas Treatment Improved the Learning and Memory Abilities in DM Rats

**FIGURE 5:**
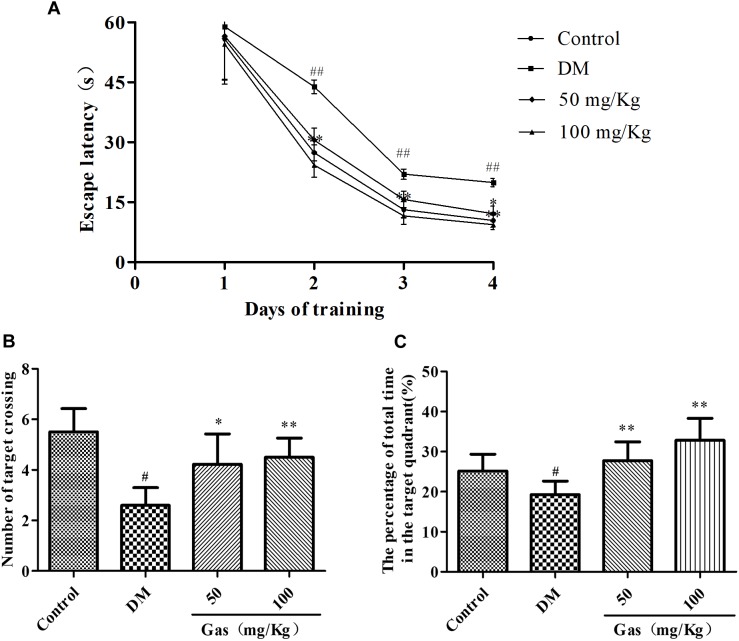
Gas ameliorates cognitive dysfunction in DM rats. **(A)** Escape latency of the 4-day hidden-platform test. **(B)** Number of target crossing in the probe trial. **(C)** Percentage of total time spent in target quadrant in the probe trial. All data are expressed as means ± SD for 10 rats in each group. ^#^*P* < 0.05, ^##^*P* < 0.01, compared with the control group. ^∗^*P* < 0.05, ^∗∗^*P* < 0.01, compared with the DM group.

To demonstrate whether Gas could improve memory impairment in DM rats, the MWM test was conducted. All groups during the four training days exhibited a decrease in the escape latency. As shown in Figure 5A, no significant differences were observed in all groups on the first training day. DM rats showed obviously higher escape latency on day 2, 3, 4 during training trials compared with those of normal rats, suggesting a significant deficits in spatial learning function (*P* < 0.01). Treatment with Gas could significantly reduce the escape latency of DM rats from training day 2 and onward (*P* < 0.05, *P* < 0.01). 24 h after the training test, a probe test was conducted on the fifth day. In the probe test, as shown in Figures 5B,C, the DM rats showed a dramatic decrease in the number of target crossing and the time spent in the target quadrant when compared with those in the control group, respectively, from 5.5 to 2.6, 25.14 to 19.25%, indicating impaired memory in DM rats (*P* < 0.05). Conversely, Gas might improve the memory dysfunction in DM rats, 50 mg/Kg Gas could increase the percentage of time spent in the target quadrant to 27.73% and the number of target crossing to 4.22, and 100 mg/Kg Gas could increase the percentage of time spent in the target quadrant to 32.86% and the number of target crossing to 4.5 (*P* < 0.05, *P* < 0.01).

### HE and Nissl Staining

**FIGURE 6:**
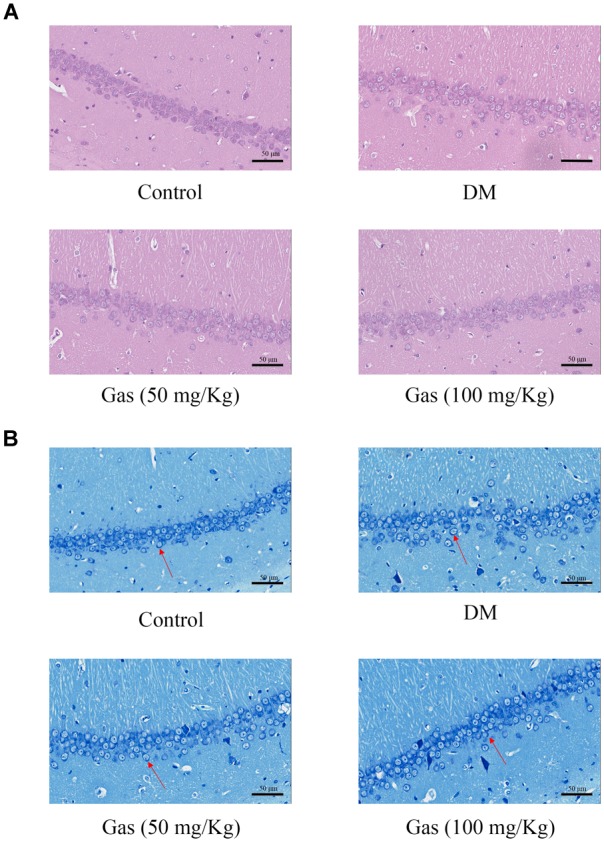
Gas prevented neuronal apoptosis in the hippocampus of DM rats. **(A)** HE staining in the hippocampal CA1 region for each group. **(B)** Nissl staining in the hippocampal CA1 region for each group.

Hematoxylin and eosin staining was used to measure protective effects of Gas on neuronal loss and damage. As shown in Figure 6A, intact neurons could be clearly seen in HE staining of control group. In DM rats, nuclei pyknosis and damaged neurons could be observed in the CA1 region of hippocampus. After Gas treatment (50 or 100 mg/Kg), nuclei pyknosis were reduced in this region of hippocampus in DM rats. The Nissl staining of the hippocampal CA1 area in brain sections was utilized to examine whether neurons were damaged in DM rats. In Figure 6B, many pyknotic neurons could be seen with scattered disorderly in the hippocampal CA1 region of DM rats. Neurons also showed light staining, indicating that cells were damaged, as multiple Nissl bodies were disappeared in neurons. However, in Gas group, dark staining of neurons could be observed in the CA1 region of hippocampus, and neurons were arranged orderly in this region. Thus, Gas can improve hippocampal damage in DM rats.

### Gas Treatment Decreased Levels of IL-1β and TNF-α in the Hippocampus

**FIGURE 7:**
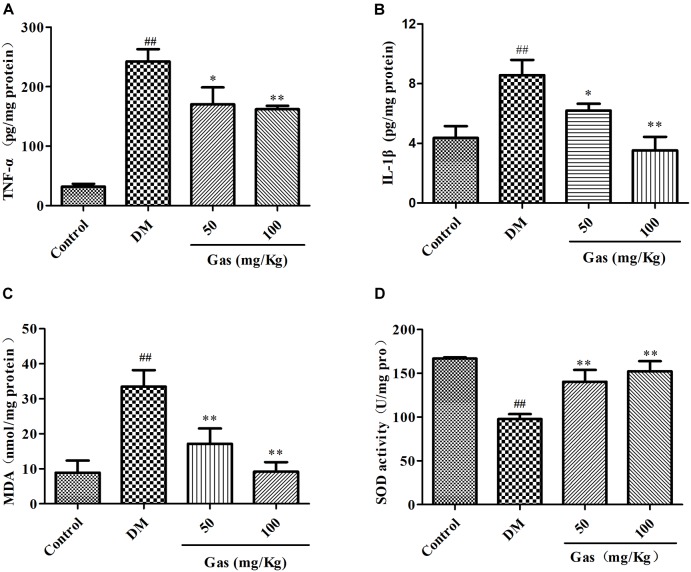
Gas decreased the contents of TNF-α, IL-1β, MDA and increased SOD contents in the hippocampus of DM rats. **(A)** Levels of TNF-α in the hippocampus of every groups after 5 weeks of treatment. **(B)** Levels of IL-1β in the hippocampus of every groups after 5 weeks of treatment. **(C)** Levels of MDA in the hippocampus of every groups after 5 weeks of treatment. **(D)** Levels of SOD in the hippocampus of every groups after 5 weeks of treatment. Values are represented as means ± SD for 10 rats in each group. ^##^*P* < 0.01, compared with the control group. ^∗^*P* < 0.05, ^∗∗^*P* < 0.01, compared with the DM group.

It has been well documented that inflammation was very crucial in DM-induced cognitive dysfunction. Therefore, we measured the amounts of IL-1β and TNF-α in the hippocampus. In Figures 7A,B, we can see that in the hippocampus of DM rats, the amount of IL-1β was increased from 4.36 pg/mg protein to 8.58 pg/mg protein, and TNF-α was increased from 31.9 pg/mg protein to 242.60 pg/mg protein (*P* < 0.01). Gas treatment for 5 weeks could significantly reduce the levels of these two indicators (*P* < 0.05, *P* < 0.01), indicating that Gas might improve the inflammatory reaction in the hippocampus of DM rats.

### Gas Treatment Improved Hippocampal Oxidative Stress in DM Rats

Chronic hyperglycemia could induce oxidative stress in the hippocampus, we measured the contents of oxidative stress markers SOD and MDA. In Figures 7C,D, SOD activity dramatically decreased from 166.94 U/mg protein to 97.79 U/mg protein, however, the amounts of MDA increased from 8.88 nmol/mg protein to 33.50 nmol/mg protein in the hippocampus of DM rats when compared with control group (*P* < 0.01). After Gas treatment for 5 weeks, SOD activity was enhanced and MDA activity was lowered in the hippocampus of DM group (*P* < 0.01). Gas could suppress hippocampal oxidative stress in DM rats.

### Gas Treatment Increased GLUT3 Expression in DM Rats

**FIGURE 8:**
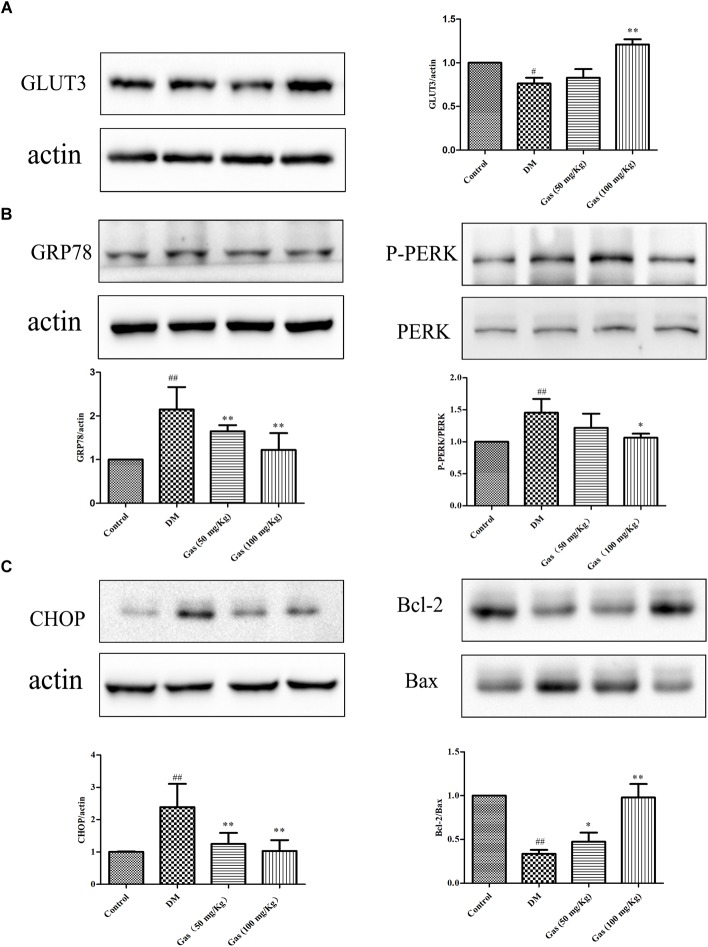
Gas increases GLUT3 expression and inhibits ERS pathway in the hippocampus of DM rats. **(A)** Representative protein bands and Western blot analysis of GLUT3 in the hippocampus of each group. **(B)** Representative protein bands and Western blot analysis of GRP78, P-PERK, PERK, CHOP, Bax and Bcl-2 in the hippocampus of each group. **(C)** Representative protein bands and Western blot analysis of CHOP, Bax and Bcl-2 in the hippocampus of each group. Values are represented as means ± SD for three rats in each group. ^#^*P* < 0.05, ^##^*P* < 0.01, compared with the control group. ^∗^*P* < 0.05, ^∗∗^*P* < 0.01, compared with the DM group.

To ensure the cognition and memory function, the brain needs constant energy supplement. GLUT3 can transport glucose from extracellular into the neurons. To evaluate the expression of hippocampal GLUT3 protein, we measured it by quantitative Western blot (*n* = 3/each group). As shown in Figure 8A, GLUT3 expression level in DM rats was decreased to 76% of the control group (*P* < 0.05). Gas treatment for 100 mg/Kg could obviously increase GLUT3 expression to about 1.51 times of the DM rats (*P* < 0.01).

### Gas Treatment Suppressed ERS and Alleviated Apoptosis in the Hippocampus of DM Rats

To further confirm the mechanisms of ERS in the pathogenesis, the GRP78 and the ERS sensors PERK, P-PERK were measured. As shown in Figure 8B, the expression of GRP78 in the DM rats was 2.15 times of the control rats. Gas treatment could reduce GRP78 expression to some extent (*P* < 0.01). When compared to the control group, the ratio of P-PERK/PERK was significantly increased to 1.43, but 100 mg/Kg Gas treatment could reduce its ratio to the normal level (*P* < 0.05). The expression of CHOP is the downstream of ERS, revealing the apoptotic condition of neurons. As shown in Figure 8C, CHOP was significantly increased to 2.4 times of the control rats (*P* < 0.01), this effect could be reversed by different dose of Gas (*P* < 0.01). Bcl-2/Bax ratio in DM rats was decreased to 30% of the healthy subjects (*P* < 0.01). After treated with Gas for 5 weeks, its ratio was remarkably increased (*P* < 0.05, *P* < 0.01).

### Gas Treatment Reduced NLRP3 Inflammasome Activation and Oxidative Stress in the Hippocampus of DM Rats

**FIGURE 9:**
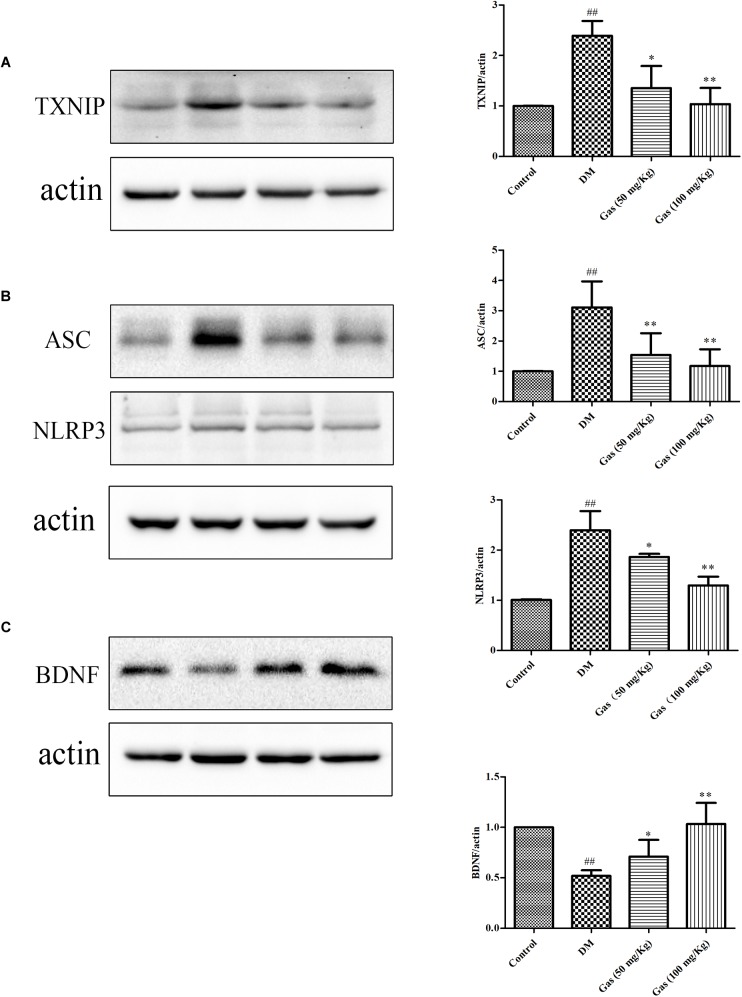
Gas inhibits TXNIP, NLRP3 inflammasome activation and increases expression of BDNF in the hippocampus of DM rats. **(A)** Representative protein bands and Western blot analysis of TXNIP in the hippocampus of each group. **(B)** Representative protein bands and Western blot analysis of NLRP3, ASC in the hippocampus of each group. **(C)** Representative protein bands and Western blot analysis of BDNF in the hippocampus of each group. Values are represented as means ± SD for three rats in each group. ^##^*P* < 0.01, compared with the control group. ^∗^*P* < 0.05, ^∗∗^*P* < 0.01, compared with the DM group.

To confirm the effects of Gas on inflammation in DM rats, we measured the expression of NLRP3 and ASC in the hippocampus. TXNIP was measured to assess the oxidative stress level. As shown in Figures 9A,B, when compared with the normal rats, TXNIP, NLRP3, and ASC expression were obviously increased in DM rats, respectively, 2.39, 2.42, 3.11 times of the control rats, indicating that the inflammation and oxidative stress was activated in the hippocampus of the DM rats (*P* < 0.01). After Gas treatment, the expression of NLRP3, TXNIP and ASC expression levels were significantly decreased (*P* < 0.01, *P* < 0.05). So, Gas treatment could significantly reduce oxidative stress and NLRP3 inflammasome activation in the hippocampus of DM rats.

### Gas Treatment Increased BDNF Expression in DM Rats

Brain derived neurotrophic factor is related to hippocampal plasticity, we examined the expression of BDNF. As shown in Figure 9C, western blot results showed a significant decrease in the level of BDNF in DM rats, reaching 51.9% of the control rats (*P* < 0.01). Gas treatment could clearly increase its expression, and high dose of Gas could increase BDNF expression to normal level, suggesting that Gas could ameliorate neural plasticity in the hippocampus of DM rats (*P* < 0.01).

## Discussion

The present study showed that high-fat and high-sucrose diet (containing normal protein level) with STZ-induced DM rats were associated with hyperglycemia, dyslipidemia and insulin resistance. Neurons under high glucose condition could result in inflammatory response, ERS and apoptosis, subsequently induce cognitive deficits. Administration of Gas for 5 weeks could improve hyperglycemia, dyslipidemia, learning and memory deficits in DM rats, inflammatory response, apoptosis and ERS in the hippocampus were also suppressed. Gas treatment could also reduce serum TC, TG, and LDL-C in DM rats. In addition, Gas promoted the expression of GLUT3 and BDNF in the model hippocampal tissue. Therefore, these findings provide potential effects of Gas on cognitive dysfunction in DM.

Patients with DM have more risk suffering from AD and VD, with brain atrophy and cognitive deficits (Jayaraman and Pike, 2014). High glucose plays a crucial role in metabolic disorder among patients with DM and it is the main risk factor for neuronal damage. In high glucose-induced neurotoxicity, oxidative stress and apoptotic neuronal play crucial roles and finally contribute to neuronal damage (Li et al., 2017). Hippocampus is a vital neurogenic area related to learning and memory function, and diabetic alterations can influence it largely (Beauquis et al., 2010; Hussain et al., 2014). Therefore, maintaining blood glucose within a normal range is the foremost objective in the treatment of T2D (Kahn et al., 2014). STZ-induced diabetic rats were associated with high levels of FBG, TC, TG, and LDL-C, which are consistent with other animal studies (Erfani Majd et al., 2018; Shamsaldeen et al., 2018).

Studies had showed that *Gastrodia elata* Blume water extract (GEB) have a beneficial effect on insulin resistance in a high fat diet-induced rats. GEB can reduce insulin resistance by reducing fat accumulation in adipocytes, and activating fat oxidation and enhancing leptin signaling in obese rats (Park et al., 2011). Moreover, in non-obese T2D rats, GEB can improve hypothalamic insulin signaling, which is associated with enhancing whole body insulin sensitivity (Yang et al., 2016). So, this kind of herb may possess potential effects on DM. Gas is the main component of GEB, it inhibits high glucose-induced human retinal endothelial cell apoptosis by regulating the SITR1/TLR4/NF-κBp65 signaling pathway, indicating its protective effects on diabetic retinopathy (Zhang et al., 2018). However, the effects of Gas on DM are still unknown. As cognitive dysfunction in DM is associated with hippocampus, so Gas may be a potential drug on this disease. In our study, administration of Gas for 5 weeks could improve hyperglycemia and dyslipidemia in DM rats. Gas has a broad range of beneficial properties on AD, PD, cerebral ischemia/reperfusion, and other cognitive deficits, the mechanisms underlying them include modulating neurotransmitters, anti-oxidative stress, suppressing microglial activation, and anti-inflammatory (Liu et al., 2018). T2D rats are cognitively impaired (Liu et al., 2016), but after Gas treatment for 5 weeks, their performance in MWM test remarkably improved.

The exposure of neurons to high glucose or hyperglycemia is known as glucose neurotoxicity. The high density of GLUT3 in neurons is important in energy supplement and neuronal activities. When glucose is transferred from blood into the hippocampus, GLUT3 can transport glucose from extracellular into hippocampal neurons (McEwen and Reagan, 2004). GLUT3 expression is suppressed in medial prefrontal cortex of DM rats (Chen et al., 2017), but its expression in hippocampus is poorly understood. In our study, the decreased GLUT3 was evident in the hippocampus of DM rats, Gas could largely enhance GLUT3 expression in the hippocampus. The brain increases its energy uptake by increasing the expression of GLUT3 to keep normal glucose metabolism of brain. The disturb of glucose metabolism will lead to oxidative stress, inflammation and ERS (Huang et al., 2018). Based on the possibility that abnormal glucose uptake was due to GLUT3 reduction in the hippocampus, we hypothesized that ERS and NLRP3 inflammasome activation were caused by perturbing intracellular glucose metabolic homeostasis.

Inflammation is related to DM and progression of DM complications (Seto et al., 2015). Inflammation is a protective mechanism against harmful substances produced in the brain. In this study, the expression of TNF-α and IL-1β increased in the hippocampus of DM rats, but could be decreased by the administration of Gas. Studies had shown that IL-1β could accelerate the pathogenesis of neurodegenerative disease and DM complications (Peiro et al., 2017), and the increment of hippocampal IL-1β expression level is related to cognitive and emotional alterations in diabetic mice (Zhai et al., 2018). The maturation of IL-1β is mediated by NLRP3 inflammasome. AD and neuropsychiatric disorders can be ameliorated by suppression of NLRP3 inflammasome activation (Gold and El Khoury, 2015). In the present study, the hippocampus of DM rats showed high expression of NLRP3 inflammasome, suggesting that high glucose could activate NLRP3 inflammasome. Gas treatment could suppress NLRP3 activation, indicating that Gas could prevent cognitive dysfunction through NLRP3 in diabetic rats.

The activation of NLRP3 inflammasome include ROS, ion fluxes, and phagosome destabilization (Han et al., 2018). Diabetes could increase ROS generation in the hippocampus of rats (Caletti et al., 2018). Gas could reduce oxidative stress by scavenging ROS in cognitive dysfunction diseases (Li and Zhang, 2015). In the present study, Gas could decrease MDA activity and increase SOD activity in the hippocampal tissue of DM rats, suggesting that Gas could decrease oxidative stress in diabetic rats. TXNIP links ROS and NLRP3 inflammasome activation. TXNIP expression is induced by glucose and its level is pathologically elevated in diabetes (Lance Thielen, 2018). In our research, TXNIP level was increased in the hippocampus of DM rats, but remarkably decreased after treatment with Gas for 5 weeks.

Diabetes is related to hyperglycemia and hyperlipidemia, leading to ERS in many peripheral tissues such as adipose tissue, muscle, and liver (Roussel et al., 2013). These three sensors of ERS, including PERK, IRE, and ATF6, can activate CHOP expression to increase the expression of Bax and can decrease the expression of Bcl-2, leading to learning and memory ability impairment in diabetic mice (Tabas and Ron, 2011). ERS could contribute to neuronal death and cell inflammation through CHOP pathway in the hippocampus of T2D rats (Sprenkle et al., 2017), it could also activate NLRP3 inflammasome in the hippocampus of high fat diet-induced rats (Cai et al., 2016). Moreover, BDNF is synthesized in the ER as the precursor protein proBDNF, which is cleaved by proteases at synapses and is converted to mature BDNF (Cai et al., 2016), which plays a crucial role in synaptic plasticity in the hippocampus. Excessive levels of IL-1β in the hippocampal can also decrease BDNF level, and cause impairments in the synaptic plasticity (Prieto et al., 2015). In accordance with this, we found that BDNF was significantly suppressed in the hippocampus of diabetic rats, Gas could increase its expression. Our study demonstrated that ERS was observed in the hippocampus of DM rats, subsequently lead to apoptosis of neurons, and ERS can also regulate the expression of BDNF. After Gas treatment, ERS was obviously suppressed and BDNF expression was increased.

## Conclusion

In summary, hyperlipidemia and hyperglycemia in DM rats could decrease hippocampal GLUT3 protein level and increase ERS, leading to decreased BDNF expression by disturbing the ER function and positively suppressing the NLRP3 pathway. After 5 weeks of Gas administration, neuroprotective effects were observed, metabolic disorders were alleviated, and hippocampal ERS could be reduced to improve BDNF expression in DM rats.

## Ethics Statement

This study was carried out in accordance with the recommendations of the Research Ethics Committee of the Chinese Academy of Medical Sciences and Peking Union Medical College, Beijing, China (SCXK 2014-0001). The protocol was approved by the Research Ethics Committee of the Chinese Academy of Medical Sciences and Peking Union Medical College.

## Author Contributions

TY, XM, GS, and XS contributed to the conception of the study. TY wrote the paper. TY, YZ, WX, and RW collected and analyzed the data.

## Conflict of Interest Statement

The authors declare that the research was conducted in the absence of any commercial or financial relationships that could be construed as a potential conflict of interest.
